# Construction of an infectious horsepox virus vaccine from chemically synthesized DNA fragments

**DOI:** 10.1371/journal.pone.0188453

**Published:** 2018-01-19

**Authors:** Ryan S. Noyce, Seth Lederman, David H. Evans

**Affiliations:** 1 Department of Medical Microbiology & Immunology and Li Ka Shing Institute of Virology, University of Alberta, Edmonton, Alberta, Canada; 2 Tonix Pharmaceuticals, Inc., New York, New York, United States of America; Universitat Bern, SWITZERLAND

## Abstract

Edward Jenner and his contemporaries believed that his *variolae vaccinae* originated in horses and molecular analyses show that modern vaccinia virus (VACV) strains share common ancestry with horsepox virus (HPXV). Given concerns relating to the toxicity of modern VACV vaccines, we asked whether an HPXV-based vaccine might provide a superior alternative. Since HPXV may be extinct and the only specimen of HPXV that has been identified is unavailable for investigation, we explored whether HPXV could be obtained by large-scale gene synthesis. Ten large (10–30 kb) fragments of DNA were synthesized based on the HPXV sequence along with two 157 nt VACV terminal sequences, and were recombined into a live synthetic chimeric HPXV (scHPXV) in cells infected with Shope fibroma virus (SFV). Sequencing of the 212 kbp scHPXV confirmed it encoded a faithful copy of the input DNA. We believe this is the first complete synthesis of a poxvirus using synthetic biology approaches. This scHPXV produced smaller plaques, produced less extracellular virus and exhibited less virulence in mice than VACV, but still provided vaccine protection against a lethal VACV challenge. Collectively, these findings support further development of scHPXV as a novel replication-proficient smallpox vaccine.

## Introduction

There had long been speculation regarding the origins of Jenner’s *variolae vaccine* [1, 2], until sequencing data showed close similarities between VACV and a Mongolian HPXV specimen [3–5]. Jenner noted that farriers sometimes milked cows and that material from the equine disease could produce a vesicular disease in cows from which *variolae vaccinae* was derived. Loy [6] and Bell [7] subsequently showed that horsepox could vaccinate humans against smallpox and these meticulous studies raise the question of whether HPXV might still serve as a live smallpox vaccine. Most of today’s VACV stocks comprise swarms of viruses that are products of ~200 years of cultivation in animals along with selection for growth in humans, and have probably evolved far from Jenner’s virus. These replication-proficient and human-adapted VACV strains can cause myopericarditis in otherwise healthy individuals [8] and are harmful in immunocompromised populations [9]. Therefore, a safer live vaccine would permit its more widespread use. However, only one specimen of HPXV has ever been identified and sequenced [4], very little is known about its biological properties, and the disease may have been accidentally extirpated [10]. This challenge led us to explore whether a replicating HPXV could be recovered by genome synthesis and might also exhibit vaccine properties.

Gene synthesis has revolutionized the study of microbes including viruses such as ΦX174, polio, and influenza [11–13]. However, poxviruses represent special challenges because of the size (many exceed 200 kbp) and the difficulty of cloning features such as the mismatched hairpin telomeres [14, 15]. Poxviruses also cannot simply be recovered from transfected cells, as the DNA is not infectious. What has been long known is that a cell infected with one poxvirus can reactivate a second, if that second virus has been rendered inert by damaging the particles [16] or extracting the DNA [17–19]. One can also perform the reactivation in cells that support the growth of different helper and reactivated viruses and then use differences in host range to separate the two. We have previously shown that SFV-infected cells offer such an advantage while providing an environment capable of assembling VACV from virus-derived restriction fragments [19].

Here we found that a replicating synthetic-chimeric HPXV with VACV hairpins can be rescued from SFV-infected cells using chemically synthesized DNA fragments. This scHPXV produced smaller plaques and less secondary plaque formation *in vitro*. In mice, scHPXV was less virulent compared to a known vaccine strain of VACV, but still provided vaccine protection against a lethal VACV challenge. Collectively, these findings support further development of scHPXV as a novel replication-proficient smallpox vaccine.

## Materials and methods

### Viruses and cell culture

SFV strain Kasza, VACV strain Western Reserve (WR), BSC-40, HeLa, Vero, and human embryonic lung (HEL) fibroblasts were originally obtained from the American Type Culture Collection. VACV strain DPP15 is from laboratory stocks [20] and cowpox virus (strain Brighton Red) was a gift from M. Barry and came originally from D. Pickup. Buffalo green monkey kidney (BGMK) cells were obtained from Diagnostic Hybrids (Athens, OH, USA). BSC-40 and BGMK cells were propagated at 37°C in 5% CO_2_ in minimal essential medium (MEM) supplemented with L-glutamine, nonessential amino acids, sodium pyruvate, antibiotics and antimycotics, and 5% fetal calf serum (FCS; ThermoFisher Scientific). HeLa and HEL cells were propagated at 37°C in 5% CO_2_ in Dulbecco’s modified Eagle’s medium (DMEM) supplemented with L-glutamine, antibiotics and antimycotics, and 10% FCS. Vero cells were propagated at 37°C in 5% CO_2_ in DMEM supplemented with L-glutamine, antibiotics and antimycotics and 5% FCS. All of the cells tested negative for mycoplasma by polymerase chain reaction.

### Synthetic chimeric (sc) HPXV genome design

**Fig 1:**
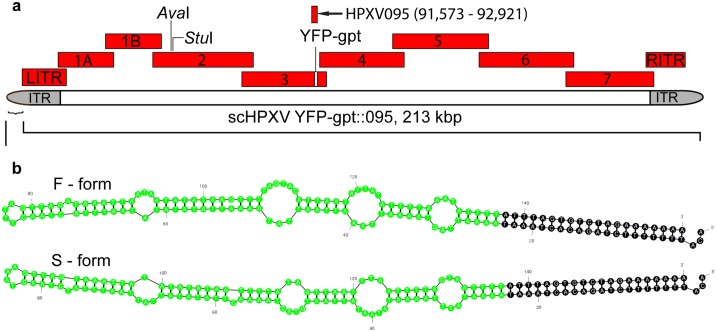
Virus design strategy. (**a**) Cloned synthetic DNA fragments used to assemble HPXV. Nine different clones were synthesized spanning all but the first and last 40 bp in GenBank entry DQ792504, each overlapping the adjacent fragment by ~1 kbp. All of the *Aar*I and *Bsa*I restriction sites were eliminated from fragments 1A to 7, inclusive, using silent mutations and the same strategy was used to add *Ava*I and *Stu*I sites in Frag_2. To facilitate virus recovery a gene encoding a YFP-gpt fusion protein was inserted into Frag_3, at the site of the HPXV thymidine kinase locus. An additional HPXV095 fragment spans the thymidine kinase locus and was subsequently used to delete and replace the YFP-gpt marker using homologous recombination. (**b**) Synthetic hairpin telomeres. Because the HPXV genome was not sequenced to the ends, we substituted two hairpin sequences based upon those reported for VACV strain WR (green coloured nucleotides). These are called “fast” and “slow” forms based upon their electrophoretic properties. The nucleotides coloured in black come from the HPXV genome sequence and provide an element essential for telomere resolution.

The design of the scHPXV genome was based on the published sequence for HPXV strain MNR-76 [GenBank accession DQ792504] [4]. We divided the 212,633 bp genome into ten fragments (Fig 1a) sharing at least 1.0 kbp of overlapping sequence with each adjacent fragment (Table 1). This provides sufficient homology to support recombination between the co-transfected fragments [19]. The terminal 40 bp from the HPXV sequence (5’-TTTATTAAATTTTACTATTTATTTAGTGTCTAGAAAAAAA-3’) was not included in the synthetic ITR fragments. Standard cloning and ligation reactions were used to ligate the VACV terminal hairpins onto the ITRs (described below).

**Table 1:** Size and location of each synthetic DNA fragment within HPXV (Accession #DQ792504).

Fragment	Size (bp)	Location (DQ792504)
LITR	10,095	41–10,135
Frag_1A	16,257	8,505–24,761
Frag_1B	16,287	23,764–40,050
Frag_2	31,946	38,705–70,650
Frag_3	25,566	68,608–94,173
Frag_4	28,662	92,587–121,248
Frag_5	30,252	119,577–149,828
Frag_6	30,000	147,651–177,650
Frag_7	28,754	176,412–205,165
RITR	8,484	204,110–212,593

Each fragment was chemically synthesized and sub-cloned into a plasmid flanked by *Sfi*I restriction sites. To aid assembly and provide a mechanism to distinguish scHPXV from other orthopoxviruses, the *Aar*I and *Bsa*I restriction sites were silently mutated in all of the fragments (S1 Table), except for the two ITR-encoding fragments (Fig 1). We chose not to mutate the *Bsa*I restriction sites in the ITRs (and there are no native *Aar*I sites), in case these regions contain sequence-specific recognition sites that serve other unknown purposes beyond protein coding.

A cassette encoding a protein consisting of yellow fluorescent protein fused to guanine phosphoribosyltransferase protein (YFP-gpt) and under the control of a poxvirus early-late promoter [21] was introduced into the HPXV095 (thymidine kinase) locus within GA_Fragment_3, so that any reactivated viruses could be detected using fluorescence. We also used silent single nucleotide substitutions to create two new restriction sites in the HPXV044 gene sequence, at positions 44,512 (*Ava*I) and 45,061 (*Stu*I), for future use as potential cloning sites.

### Preparation of HPXV DNA fragments

All of the HPXV DNAs were synthesized by GeneArt and supplied as plasmid clones in *E*. *coli*. The HPXV fragments were digested free of the cloning vectors and purified using a QiaexII kit in preparation for transfection. The Slow (S) and Fast (F) forms of the VACV terminal hairpin loops were purchased from Integrated DNA Technologies as 157 nucleotide DNA fragments. These hairpin ends were added to the left and right ITR fragments, in a random orientation using DNA ligase and in a manner that retained all of the original published HPXV sequence. Agarose gel electrophoresis was used to demonstrate that the VACV terminal hairpins were successfully added to the cloned ITR fragments (S1 Fig).

### Reactivation of VACV/HPXV hybrid viruses in SFV-infected cells

The infection and transfection reactions were performed as described previously using SFV-infected BGMK cells [19]. The Lipofectamine 2000 complexes (ThermoFisher Scientific) contained ~0.5 μg of each of the digested HPXV DNA fragments along with 0.5 μg of purified VACV WR genomic DNA. One day after transfecting the cells, the media was replaced with fresh MEM containing 5% FCS and the cells returned to the incubator for four more days. The viruses were harvested by freeze thaw, diluted and re-plated on BSC-40 cells under 0.9% agar, and any fluorescent plaques plaque purified at least three times. To rapidly confirm the presence of HPXV DNA in the reactivated viruses, PCR primers were designed flanking all of the *Bsa*I sites disrupted in the HPXV clones (S2 Table), but which are retained at the homologous sites in VACV. Virus DNA was extracted from three different hybrid (i.e. fluorescent) viruses, as well from VACV, subjected to PCR amplification, and digested with *Bsa*I. The reaction products were analyzed by agarose gel electrophoresis using SYBR^®^ safe stain to visualize DNA.

### Reactivation of scHPXV in SFV-infected cells

The infection and transfection reactions were performed as described above except using 5 μg total HPXV DNA fragments, in equimolar ratios, and omitting the VACV DNA. After an additional four days of culture in replenished MEM, the infected cells were scraped into the culture medium and the virus released using three cycles of freeze-thaw. The cell extract was diluted in serum-free MEM and aliquots plated on BSC-40 cells. One hour post infection, the inoculum was replaced with MEM containing 5% FCS and 0.9% noble agar and cultured for five days. Fluorescent plaques were visualized under an inverted microscope and individual plaques were picked for further analysis. These scHPXV YFP-gpt::095 viruses were subsequently plaque purified three times using yellow fluorescence as the selection criterion. Three different clones were retained from three separate transfections and annotated as “plaque picks” 1-to-3 (i.e. PP1.1, PP2.1, and PP3.1)

### Restriction digestion and pulse-field gel electrophoresis (PFGE) analysis of scHPXV

The viruses were purified using sucrose gradients and genomic DNA prepared as described [22]. For pulse-field gel electrophoresis (PFGE), 100ng of viral genomic DNA was digested with *Bsa*I or *Hind*III for 2h at 37°C and separated using 1% Seakem Gold agarose gels, cast and run in half-strength Tris-borate-EDTA electrophoresis buffer. The DNA was resolved on a CHEF DR-III apparatus (BioRad) at 5.7V/cm for 9.5h at 14°C, using a switching time gradient of 1 to 10s, a linear ramping factor, and a 120° angle. This separates DNAs from 1kbp to >200kbp. The DNA was visualized with SYBR^®^ gold stain.

### Virus DNA isolation, genome sequencing, and analysis

Virus DNA was extracted from each of eight sucrose-gradient purified virus preparations using proteinase K followed by phenol-chloroform. The amount of DNA was determined using a Qubit dsDNA HS assay kit (ThermoFisher Scientific) and then each genome was sequenced at the University of Alberta. Sequencing libraries were generated using a Nextera Tagmentation kit (Epicentre Biotechnologies) and paired end sequencing (2x300bp) performed using an Illumina MiSeq platform with an average read depth of 3,100 reads·nt^-1^ across the genome and ~190 reads·nt^-1^ in the F- and S-hairpins. The raw sequence reads were trimmed and initially mapped to the HPXV reference sequence using CLC Genomics Workbench 8.5 software. All nucleotide insertions, deletions, and substitutions within the scHPXV YFP-gpt::095 sequence were verified against the HPXV reference sequence. Genome Annotation Transfer Utility software (GATU) [23] was used to transfer the reference annotation to our assembled genome sequences.

### Production of a wild-type recombinant scHPXV

The YFP-gpt selection marker was removed using homologous recombination. To do this, we synthesized a 1349 bp DNA fragment encoding HPXV nucleotides 91573–92921 (ThermoFisher Scientific) and encompassing the HPXV095 gene plus ~400 bp of homology flanking either side of the thymidine kinase locus (Fig 1). This DNA was cloned into a commercial vector provided by GeneArt. To replace the YFP-gpt cassette, BSC-40 cells were infected with scHPXV YFP-gpt::095 and then transfected with 2 μg of linearized plasmid using Lipofectamine 2000 (ThermoFisher Scientific). The virus progeny were harvested two days later and the non-fluorescent viruses were isolated using three rounds of plaque purification under agar. The PCR and primers that targeted sequences flanking the HPXV095 gene locus (5’-CCTATTAGATACATAGATCCTCGTCG-3’ and 5’-CGGTTTATCTAACGACACAACATC-3’) were used to show that we had restored the wild-type locus. The scHPXV genome sequence containing the VACV F- and S-terminal hairpins arbitrarily placed at the left and right termini of the genome, respectively, has been deposited in GenBank as accession number KY349117.

### Virus growth properties and plaque size measurements

To measure virus growth, near confluent monolayers of BSC-40, HeLa, HEL fibroblasts, and Vero cells were infected at 0.01 PFU/cell with the indicated viruses. The cells were harvested at different time points and the yield of infectious particles was determined by plaque assay on BSC-40 cells. To measure plaque size, BSC-40 cells were infected for 3 days with the indicated viruses at 50 PFU/60mm dish and then the cells were fixed and stained with crystal violet. A digital image of each dish was obtained and the plaque areas were determined using FIJI software [24]. In each condition at least 8 plaques per virus were measured in three independent experiments.

### Murine intranasal model of scHPXV infection

Six-to-eight week old BALB/c mice were purchased from Charles River Laboratories. Groups of five mice were intranasally infected (or mock infected) with 5×10^3^ PFU VACV (strain WR), 1×10^7^ VACV (strain DPP15 [20]), or 1×10^5^ PFU, 1×10^6^ PFU, or 1×10^7^ PFU of scHPXV YFP-gpt::095 or scHPXV in 10μl of PBS. The mice were weighed daily for 28 days, and the following clinical signs were scored: ruffled fur, difficulty breathing, reduced mobility, and pox lesions. All of these mice were subsequently challenged intranasally with 1 × 10^6^ PFU VACV (strain WR) and monitored daily as described above for 14 days. Mice that lost 25% of the initial weight were euthanized.

For plaque reduction and neutralization tests, blood was collected from each of the mice via the saphenous vein at day 16 post-vaccination and equal quantities were pooled within each cohort. The serum was heated at 56°C for 30 min and then 2-fold serial dilutions prepared in serum-free media. The diluted serum (50μL) was mixed with 50 μL of serum-free media containing ~75 pfu of VACV strain WR, incubated at 37°C for 60 min, and then plated on BSC-40 cells. The plaques were counted two days later and the PRNT_50_ estimated from the interpolated dilution that would produce a 50% reduction in virus titer.

### Biosafety, biosecurity and animal care

All of the studies concerning the reconstruction of HPXV were conducted with the knowledge and approval of the University of Alberta Biosafety Officers. All work was performed under Containment Level 2 physical and operational conditions in compliance with the *Canadian Biosafety Standard* as published by the Public Health Agency of Canada and the Canadian Food Inspection Agency of Canada, with the additional voluntary precaution of prohibiting researchers' contact with horses or cattle. As part of the institution's *Human Pathogens and Toxins Act* license to work with pathogens, the University of Alberta submitted a plan detailing how it administratively manages and controls biosafety and biosecurity risks, which was considered acceptable by the Public Health Agency of Canada. These ongoing efforts were also disclosed to the World Health Organization (WHO) through DE’s participation in activities relating to the Advisory Committee on Variola Virus Research. All of the animal experiments were reviewed and approved by the University of Alberta Animal Care and Use Committee and Research Ethics Office.

## Results

### scHPXV design strategy

Given that the toxicity of modern VACV vaccines limits their use, and molecular analyses show that modern VACV vaccine strains share common ancestry with HPXV, we asked whether a HPXV-based vaccine might provide a safer alternative. Using the HPXV (strain WNR-76) sequence [4] we divided the 212,633 bp genome into 10-overlapping DNAs sharing ~1 kbp of flanking homology (Fig 1). Each fragment was synthesized and cloned into a bacterial vector. To facilitate the assembly of these clones we deleted all of the *Aar*I or *Bsa*I sites in fragments 1–7 using silent mutations, but did not mutate the *Bsa*I sites in the two inverted terminal repeat (ITR) fragments, as these regions contain elements required for genome replication and resolution. In addition we used silent substitutions to introduce *Ava*I and *Stu*I sites into the HPXV044 gene and replaced HPXV095 in Frag_3 with sequences encoding yellow fluorescent protein fused to guanine phosphoribosyltransferase protein (YFP-gpt) [21]. HPXV095 encodes the HPXV homolog of the non-essential VACV J2R gene.

**Fig 2:**
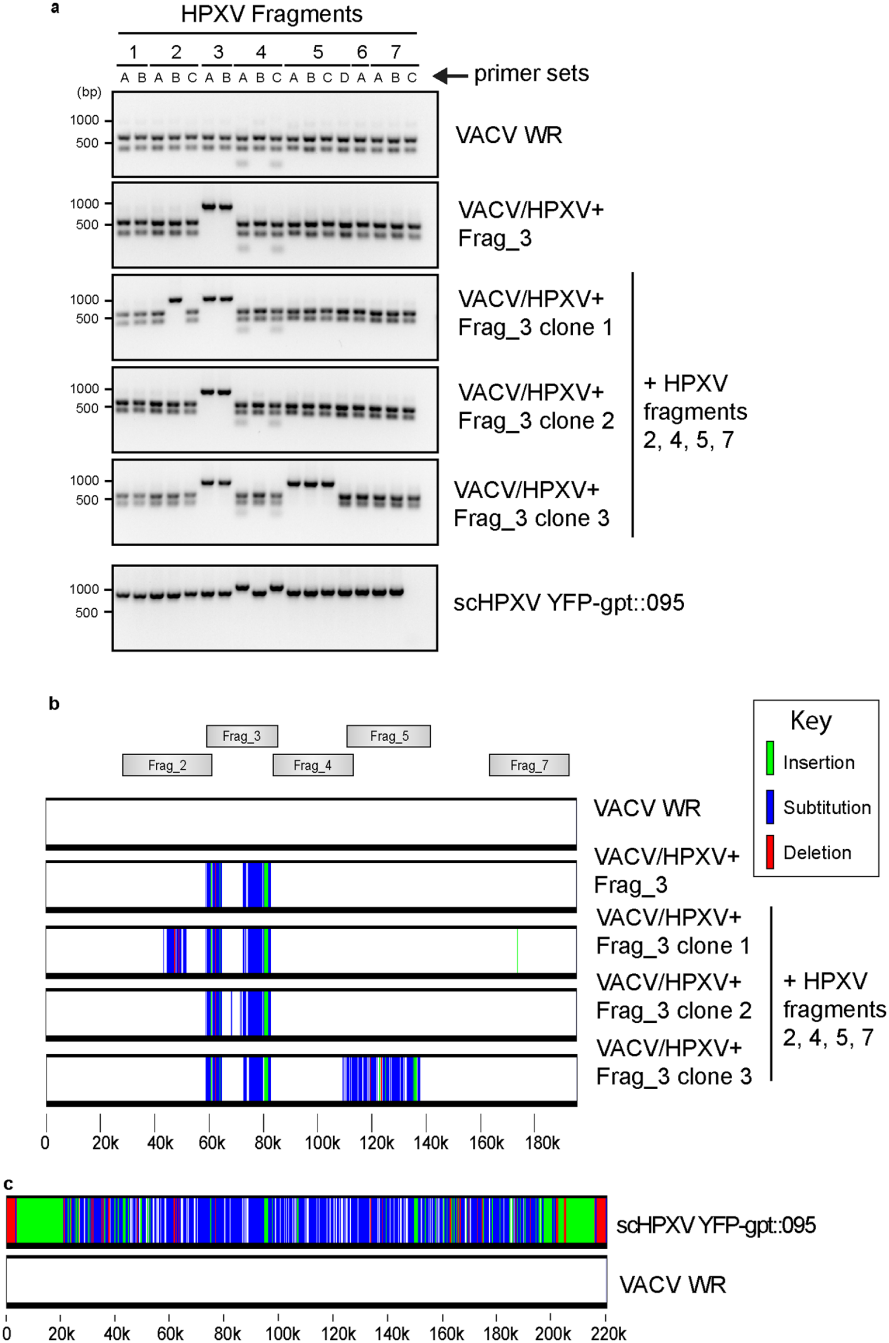
Characterization of VACV-HPXV hybrid viruses. **a**. A PCR-based screening approach was used to identify hybrid and reactivated viruses. PCR primers were designed to target both HPXV and VACV (S2 Table) and used to amplify DNA segments spanning the *Bsa*I sites that were disrupted in the synthetic HPXV clones. Following PCR amplification, the products were digested with *Bsa*I to differentiate VACV sequences (which cut) from HPXV (which do not cut). The VACV/HPXV hybrids exhibit a mix of *Bsa*I sensitive and resistant sites whereas a reactivated scHPXV YFP-gpt::095 clone is fully *Bsa*I resistant. **b**. Sequence mapping of HPXV inserts in VACV strain WR. Virus genomes were sequenced using an Illumina platform, assembled, and LAGAN [26] and “Base-by-Base” [27] software were used to align and generate the maps shown. Places where VACV sequences (white) have been replaced by HPXV sequences are colour coded according to the difference. The first hybrid virus (“VACV/HPXV + fragment 3”) was obtained by co-transfecting VACV DNA plus HPXV Frag_3 (Fig 1) into SFV-infected cells. The green-tagged insertion encodes the YFP-gpt selection marker. Clones 1–3 were obtained by purifying the DNA from this first hybrid genome and transfecting it again, along with HPXV fragments 2, 4, 5, and 7, into SFV-infected cells. **c**. Genomic sequence comparison of scHPXV YFP-gpt::095 to VACV WR.

As a proof of principle, co-transfection of HPXV Frag_3 along with VACV genomic DNA into SFV-infected BGMK cells led to the recovery of a hybrid virus (VACV/HPXV+Frag_3), validating the selection strategy (Fig 2). The addition of other HPXV synthetic DNA fragments along with genomic DNA from this hybrid virus, however, did not result in complete incorporation of the HPXV fragment lacking *Bsa*I sites into the genomes of these hybrid virus clones (Fig 2a). Instead, these viruses exhibited a “patchy” pattern of DNA integration, similar to what is detected in virus-by-virus crosses (Fig 2b) [25]. This patchy pattern is easily detected because of the sequence polymorphisms that differentiate HPXV from VACV. These sequence differences are illustrated by an alignment of the two viruses (Fig 2c).

The HPXV ITRs presented more challenges, because the available HPXV genome was not sequenced through the hairpin ends. The HPXV sequence stops at a feature needed for concatemer resolution [28, 29], which in VACV is located ~60 nt from the end of the genome and it is identical to the putative resolution site in the HPXV sequence. Given the similarities between the two viruses, we tested whether VACV hairpins could substitute for the missing HPXV ends. The fast [F] and slow [S] VACV hairpins (Fig 1b) were synthesized and these two 157 nt oligonucleotides were ligated onto the left and right HPXV ITR fragments using standard molecular biology techniques. This produced four combinations of ITR-hairpin species with joints that copied the desired chimera and with no adventitious sequences (S1 Fig). We pooled these DNAs in reactivation reactions.

**Fig 3:**
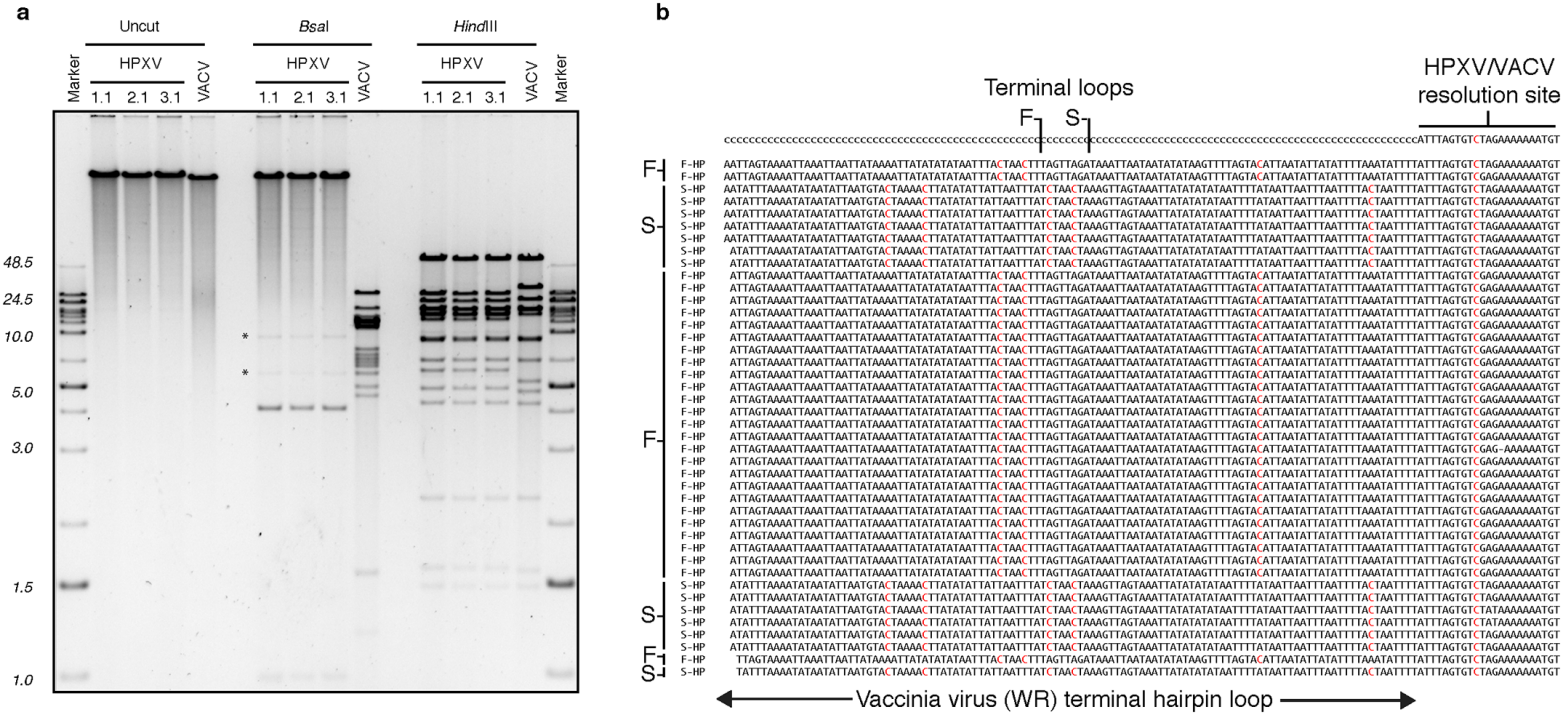
Sequencing and restriction endonuclease mapping of reactivated scHPXV YFP-gpt::095 clones. **a**. Pulsed field gel electrophoretic analysis. Three independent HPXV clones plus a VACV (strain WR) control virus were purified and then left either untreated, or digested with *Bsa*I or *Hin*dIII. The larger size of HPXV relative to VACV is apparent in the uncut samples, and the absence of nearly all of the *Bsa*I sites in the HPXV clones is also apparent. The selective manner in which only the *Bsa*I sites were modified by gene synthesis is illustrated by the retention of all the *Hin*dIII restriction sites in scHPXV YFP-gpt::095. The *Hin*dIII digest also demonstrates the similarities between HPXV and VACV as illustrated by the related digestion patterns. The faint DNA bands marked with asterisks (*) are from monkey cell mitochondria, which co-purify with poxvirus particles. **b**. Sequence reads associated with the hairpin telomeres. The viruses were sequenced using Illumina technology and the genomes assembled using CLC genomics software. A subset of the longest sequencing reads, extending beyond the known end of the HPXV sequence, are shown aligned against a poly·dC template. (This method captures sequences extending beyond the point where the reference sequence ends.) These reads span the entire length of the unfolded hairpins as was provided using synthetic oligonucleotides. Because of the inverted terminal duplications, all of the reads “pile up” together. Both F- and S-forms of the VACV hairpin are detected and the ratio of F- to S-reads in this region was 1.03±0.01 (SEM) in eight different virus-sequencing reactions.

We then transfected an equimolar mix of all ten HPXV fragments into SFV-infected cells to test whether these synthetic HPXV fragments could be recombined into replication-competent viruses. Five days later, the virus mix was harvested and re-plated on BSC-40 cells, where Orthopoxviruses will form plaques. Although this process is inefficient compared to transfection of complete poxvirus genomes into cells where we and others can get up to 10^6^ pfu/μg [18, 19], we obtained from 0-to-4 fluorescent plaques on the BSC-40 cells, in each of six replicate experiments, or ~30 pfu/μg of transfected DNA. Judging by pulse field gel electrophoresis these viruses appeared to be chimeras that encoded synthetic HPXV sequences bounded by HPXV/VACV hairpin ends (Fig 3a). We sequenced five viruses from two reactivation reactions and all faithfully replicated the sequence of the input clones. We confirmed that this method doesn't introduce SFV DNA into the reactivated viruses [25] and the only mutations detected were those we had incorporated by design, for example novel *Ava*I and *Stu*I sites in HPXV044 (S2 Fig). Our Illumina sequencing also detected reads extending completely through the VACV-derived hairpin telomeres. The 1:1 distribution of F- and S-reads in each of the five reactivated viruses suggests that both ends are required to produce a virus (Fig 3b and S3 Table). However, because of the way that these sequences are produced by fragmentation of a pool of viruses bearing inverted terminal repeats, it is not possible to establish how they are oriented or distributed with respect to any particular individual genome.

We subsequently synthesized a 1,349 bp DNA encoding the HPXV095 locus along with flanking sequences (Fig 1a) and transfected it into cells infected with one of the reactivated HPXV. Three non-fluorescent recombinant viruses were recovered and purified, and PCR and sequencing were used to confirm that we had restored the wild-type HPXV095 gene. This “synthetic and chimeric” virus was labeled scHPXV to differentiate it from the reactivated thymidine kinase deficient parent virus (scHPXV YFP-gpt::095) and the native virus (HPXV strain WNR-76).

### scHPXV growth properties *in vitro*

**Fig 4:**
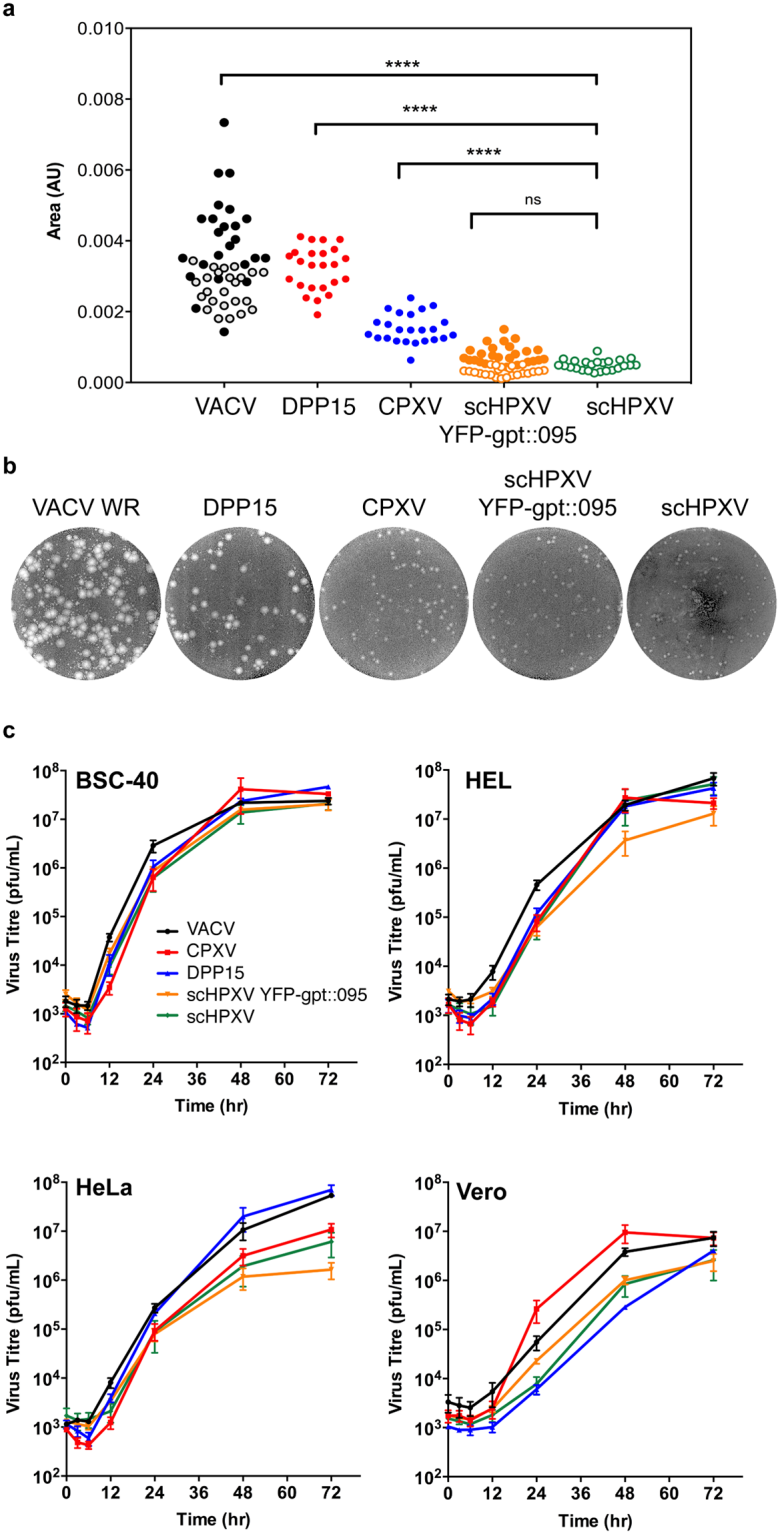
scHPXV YFP-gpt::095 and scHPXV growth properties in culture. **a**. Plaque size measurements. BSC-40 cells were infected with the indicated viruses and cultured for three days. The dishes were stained and the plaque areas measured using a scanned digital image. Approximately 8 plaques were measured per virus treatment from three independent infections (closed circles). A similar independent experiment comparing scHPXV YFP-gpt::095 and scHPXV to VACV WR was also completed as described above (open circles). DPP15 is a VACV clone isolated from Dryvax vaccine and CPXV is cowpox virus (strain Brighton Red). scHPXV YFP-gpt::095 and scHPXV form plaques that are significantly smaller than any of the other two viruses tested. Statistically significant differences are noted (****P^*2*^ 0.0001). **b**. Plaque morphology seen at three days post-infection on BSC-40 cells. The cells were fixed and stained with crystal violet. **c**. Multi-step growth kinetics measured using human (HEL, HeLa) or monkey (BSC-40, Vero) cells. The cells were infected at a multiplicity of infection of 0.01, the virus harvested at the indicated times, and the virus titrated on BSC-40 cells in triplicate. The error bars indicate the SEM.

There are no publications that extensively compare the properties of HPXV with other Orthopoxviruses. We used VACV strain Western Reserve, DPP15 (a VACV Dryvax^™^ clone resembling ACAM2000 [20]), and cowpox strain Brighton for comparison purposes. The most obvious difference related to plaque size, scHPXV YFP-gpt::095 and scHPXV formed plaques that are significantly smaller than other Orthopoxviruses on BSC-40 cells (Fig 4a and 4b). The scHPXV small plaque phenotype appears identical to what has been reported for authentic HPXV [30]. Different VACV strains also produce extracellular viruses that form smaller secondary plaques [31]. These are not produced by scHPXV strains (Fig 4b) and the reasons for this difference are not obvious since all of the known genes regulating extracellular enveloped virus production [31] are found in HPXV. Interestingly the small plaque phenotype is not reflected in multi-step growth curves, at least on BSC-40 cells where both scHPXV YFP-gpt::095 and scHPXV grew as well as the other viruses. The virus grew to somewhat lower titers on primary human fibroblasts and Vero cells, and least well on HeLa cells (Fig 4c). The finding that scHPXV does not produce secondary plaques (a characteristic feature of extracellular enveloped virus producing viruses), may be of relevance given that this property affects virulence [32].

### Virulence and vaccine properties of scHPXV

**Fig 5:**
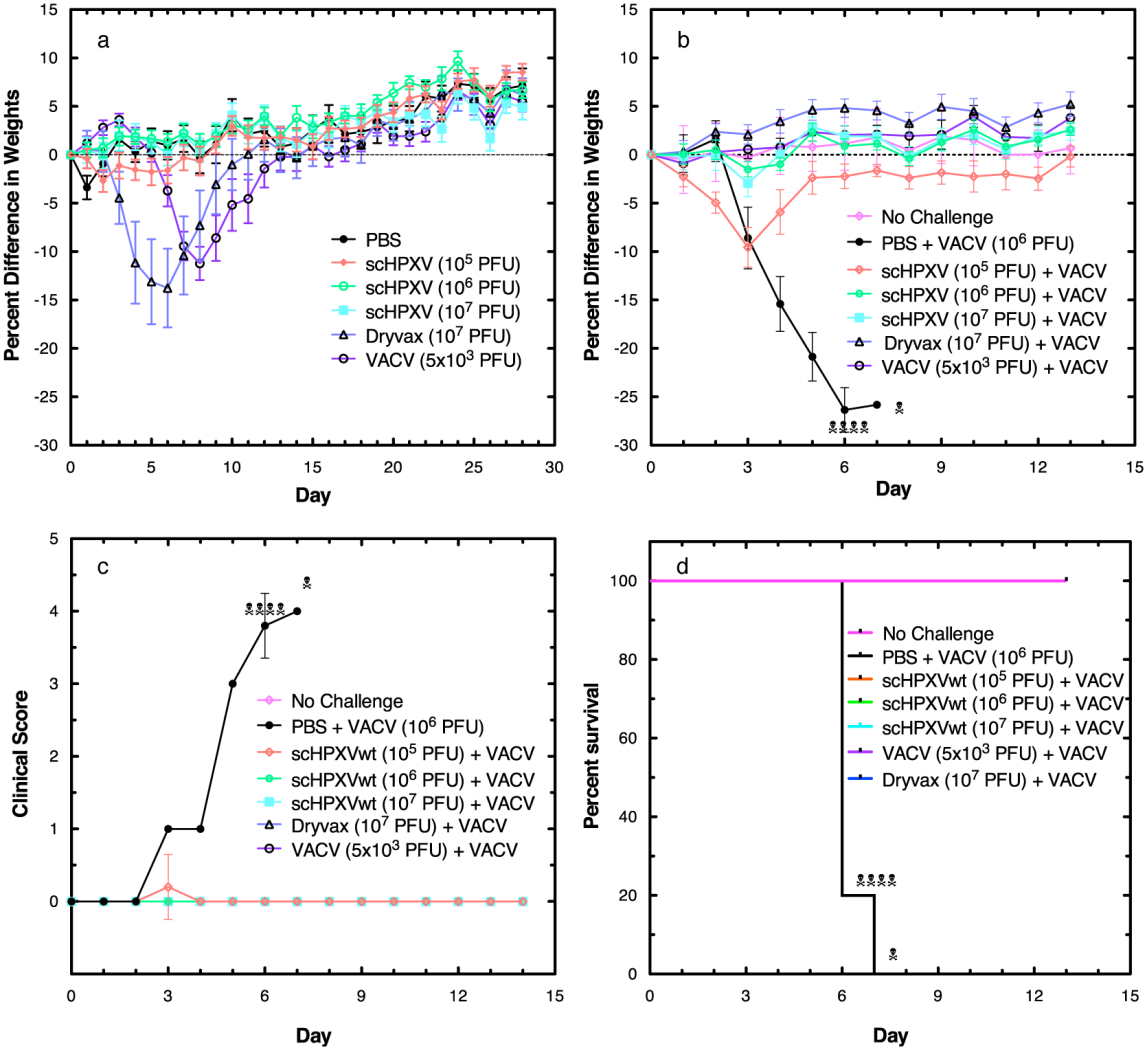
Virulence and vaccine properties of scHPXV. **a**. Virulence studies. Immune competent BALB/c mice (5 per group) were inoculated intranasally with the indicated viruses. scHPXV was tested using doses ranging from 10^5^−10^7^ pfu per mouse. Some weight loss due to illness was seen in animals inoculated with 5×10^3^ pfu VACV strain WR (¢), or 1×10^7^ pfu of VACV strain DPP15 (**Δ**), but no illness was detected in any of the animals infected with HPXV clones. **b**. VACV challenge studies. Four weeks after exposure to the indicated agent as shown in panel **a** (or mock treatment with buffered saline), the mice were exposed to a lethal dose of VACV strain WR (1×10^6^ pfu) by the same route (day 0 in **b** is day 28 in graph **a**). The animals were monitored for signs of disease and euthanized if the weight loss exceeded 25% of the initial body weight. The scHPXV strain provided good protection at the two highest pre-challenge doses (1×10^6^ and 1×10^7^ pfu). **c**. Disease course in HPXV vaccinated mice. The mice were inspected to detect four signs of disease (ruffled fur, hunched posture, difficulty breathing, and reduced mobility) and a clinical score calculated from the sum of the individual scores averaged across all five (or surviving) mice per cohort. Little or no signs of illness were detected in mice first vaccinated with 1×10^6^ or 1×10^7^ pfu scHPXV **d**. Kaplan-Meyer analysis of survivorship. All of the mock-vaccinated (i.e. saline-treated) mice succumbed to VACV strain WR challenge, whereas treatment of animals vaccinated with either VACV strain WR, VACV strain DPP15, or all doses of scHPXV (10^5^−10^7^ pfu per mouse) protected 100% of the animals.

We also tested the virulence of scHPXV and scHPXV YFP-gpt::095 in mice and whether they could protect against lethal VACV challenge. For purposes of comparison we used VACV strains WR and DPP15, which at the indicated doses, are known to cause virulence in BALB/c mice. BALB/c mice were inoculated with 10^5^-to-10^7^ pfu of each of the HPXV, 1×10^7^ pfu of VACV strain DPP15, or 5×10^3^ pfu of VACV strain WR. The mice inoculated with VACV exhibited symptoms of illness most graphically illustrated as transient weight loss and with the lower dose of VACV (WR) taking longer to elicit disease (Fig 5a). No signs of infection (e.g. weight loss, ruffled fur, or lethargy) were detected in any of the animals inoculated with either scHPXV YFP-gpt::095 (S3 Fig) or scHPXV strains (Fig 5a). All of the animals were fully recovered two weeks later. On day 16, blood samples were collected via the saphenous vein and pooled within each cohort, and titered to measure VACV-specific antibodies using a plaque reduction neutralization test (PRNT). The PRNT_50_ antibody titres in mice infected with 1×10^7^ pfu of the two HPXV ranged from 1:35 (scHPXV) to 1:70 (scHPXV YFP-gpt::095), comparable to that detected in animals infected with 1×10^7^ pfu of VACV strain DPP15 (1:20) or 5×10^3^ pfu of VACV strain WR (1:60), and above the background in controls (~1:2). All of these animals were subsequently challenged with a lethal dose of VACV (WR) at day 30 post-infection (Fig 5b–5d and S3b–S3d Fig). All of the control animals initially treated with saline had to be euthanized due to the infection within 7 days. The animals initially exposed to 1×10^7^ pfu of VACV (DPP15) or 5×10^3^ pfu of VACV (WR), or 1×10^6^ or 1×10^7^ pfu of scHPXV showed little or no weight loss and no illness as judged by the clinical scores (Fig 5c). Mice vaccinated with 10^5^ pfu of scHPXV YFP-gpt::095 were only partially protected from a lethal dose of VACV WR (S3d Fig), suggesting that mutating the thymidine kinase attenuates the virus in mice. We concluded that scHPXV can immunize against a lethal VACV challenge and without causing disease during the intranasal immunization step.

## Discussion

These studies were provoked by our interest in the history of Jenner’s vaccine. Contemporary accounts [6, 7] provide support for Jenner’s speculation that the vaccine probably originated as an equine disease called “grease”. Most notably Loy showed that one of two forms of grease (or horsepox) characterized by a diffuse pox reaction, provided protection against smallpox without requiring passage through cows [2]. Modern sequencing supports this hypothesis with the one known HPXV strain most closely resembling both old French [3] and early American [5] smallpox vaccines, and sharing a common origin with all known VACV [33]. Further support for a shared origin is provided by the observation that the I4L locus in a rare Dryvax clone (DPP17), encodes a block of sequence and a frameshift mutation that most closely resembles the homologous locus in HPXV [20]. Using the HPXV genome sequence [4], synthetic DNA fragments, and methods outlined elsewhere [34], we have used recombination and reactivation reactions to recreate this HPXV strain. This scHPXV resembles VACV in many of its growth properties, although it produces much smaller plaques (Fig 4a) without the secondary plaques associated with extracellular virus, which may partly explain the reduced virulence. Whether the increased infectivity and virulence of VACV reflects two centuries of selection in alternative hosts, including passage in humans, is an interesting question and because VACV-HPXV hybrids are viable (Fig 2), such strains might provide a tool for identifying some of the gene(s) responsible. Of perhaps most interest is the fact that our scHPXV strain generates immune protection against lethal virus challenge (Fig 5), and if the lower virulence in mice reflects better tolerability in humans, it supports further investigation as a vaccine and vector.

The method described here could be adapted to assemble other Orthopoxviruses, and possibly other Chordopoxviruses if the right helper virus(es) and supporting cell lines can be identified. This is timely as poxviruses offer much promise as oncolytic agents [35, 36] and better ways are needed to more rapidly and extensively engineer these agents to provoke potent anti-tumour immune responses; ideally using designs based on personalized cancer neo-antigens. These synthetic approaches also provide tools for investigating certain basic features of virus genome structure (for example the mismatched and flip/flop hairpin telomeres) that are still incompletely understood 3–4 decades after they were discovered [37].

That said this is clearly an example of dual use research, and observations like these poses significant challenges for public health authorities. Most viruses could be assembled nowadays using reverse genetics, and these methods have been combined with gene synthesis technologies to assemble poliovirus [12] and other extinct pathogens like the 1918 influenza strain [13]. Given that the sequence of variola virus has been known since 1993 [38], our studies show that it is clearly accessible to current synthetic biology technology, with important implications for public health and biosecurity [34]. Our hope is that this work will promote new and informed public health discussions relating to synthetic biology, stimulate new evaluation of HPXV-based vaccines, and advance the capacity to rapidly produce next-generation vaccines and poxvirus-based therapeutics.

## Supporting information

S1 Dataset (XLSX)

S1 Fig Construction of hairpin-terminated left and right ITRs. **a**. Restriction maps showing the arrangement of *Sfi*I and *Pvu*II restriction sites in the left and right ITR clones. **b**. Gel electrophoretic analysis of ligation products. The cut ITR clones were ligated with or without the fast (F) or slow (S) forms of the VACV hairpin (H.P.) oligonucleotides and then a portion of each sample was digested with *Pvu*II to aid in detecting the small size shift associated with adding hairpin ends. We observed nearly quantitative ligation of the ITR ends to the hairpin oligonucleotides as judged by the bands running at ~1.5 kbp (“*”).
(TIF)

S2 Fig Genome sequence analysis of scHPXV YFP-gpt::095. The recovered viruses were sequenced using “Tagmentation” and Illumina technologies and the genomes assembled using CLC genomics software and both “map to reference” and *de novo* approaches. Shown here are a portion (~1%) of the sequence reads that mapped to the HPXV044 gene (VACV I4L homolog) in DQ792504 (top row). Fragment_2 encoded a silent T-to-G substitution mutation at position 44,512 designed to introduce a novel *Ava*I site into the reactivated viruses (“Consensus”). Apart from the mutations that were deliberately incorporated into the synthetic sequence, like this T-to-G substitution, no other mutations were detected by genome sequencing across the scHPXV YFP-gpt::095 or scHPXV genomes.
(TIF)

S3 Fig Effect of thymidine kinase mutations on HPXV virulence and vaccine properties. **a**. Virulence studies. Immune competent BALB/c mice (5 per group) were inoculated intranasally with the indicated viruses. Both scHPXV and scHPXV YFP-gpt::095 (DJ2) were tested, at doses ranging from 10^5^−10^7^ pfu per mouse. No illness was detected in any of the animals infected with HPXV clones. **b**. VACV challenge studies. Four weeks after exposure to the indicated agents as shown in panel **a**, the mice were exposed to a lethal dose of VACV strain WR (1×10^6^ pfu) by the same route. (The “no challenge” controls were PBS treated and unchallenged.) The animals were monitored for signs of disease and euthanized if the weight loss exceeded 25% of the initial body weight. The animals previously treated with the scHPXV YFP-gpt::095 strain acquired only limited and dose-dependent protection from VACV strain WR, whereas the scHPXV strain provided good protection at the two highest pre-challenge doses (1×10^6^ and 1×10^7^ pfu). **c**. Disease course in HPXV-vaccinated mice. The mice were inspected to detect four signs of disease (ruffled fur, hunched posture, difficulty breathing, and reduced mobility) and a clinical score calculated from the sum of the individual scores averaged across all five (or surviving) mice per cohort. Little or no signs of illness were detected in mice first vaccinated with 1×10^6^ or 1×10^7^ pfu scHPXV whereas the scHPXV YFP-gpt::095 provided incomplete protection at the lower pre-challenge doses (1×10^5^ or 1×10^6^ pfu). **d**. Kaplan-Meyer analysis of survivorship. The lowest dose of scHPXV YFP-gpt::095 (1×10^5^ pfu) provided only partial protection from a VACV challenge. All other interventions protected 100% of the animals. Note that data showing the behavior of the scHPXV strain (presented in Fig 5) are also duplicated here for comparison purposes.
(TIF)

S1 Table Silent mutations created in scHPXV fragments to remove *Aar*I and *Bsa*I restriction sites from HPXV genome. (DOCX)

S2 Table Primers used to amplify regions within VACV and HPXV that flank *Bsa*I restriction endonuclease sites found in VACV (NC_006998). (DOCX)

S3 Table Read statistics of F- and S- hairpins (HP) in reactivated scHPXV clones. (DOCX)
