# Supplementary figures and images for: Construction of an infectious horsepox virus vaccine from chemically synthesized DNA fragments
Source: PLoS One. 2018 Jan 19;13(1):e0188453. doi: 10.1371/journal.pone.0188453 (PMC5774680; doi:10.1371/journal.pone.0188453)

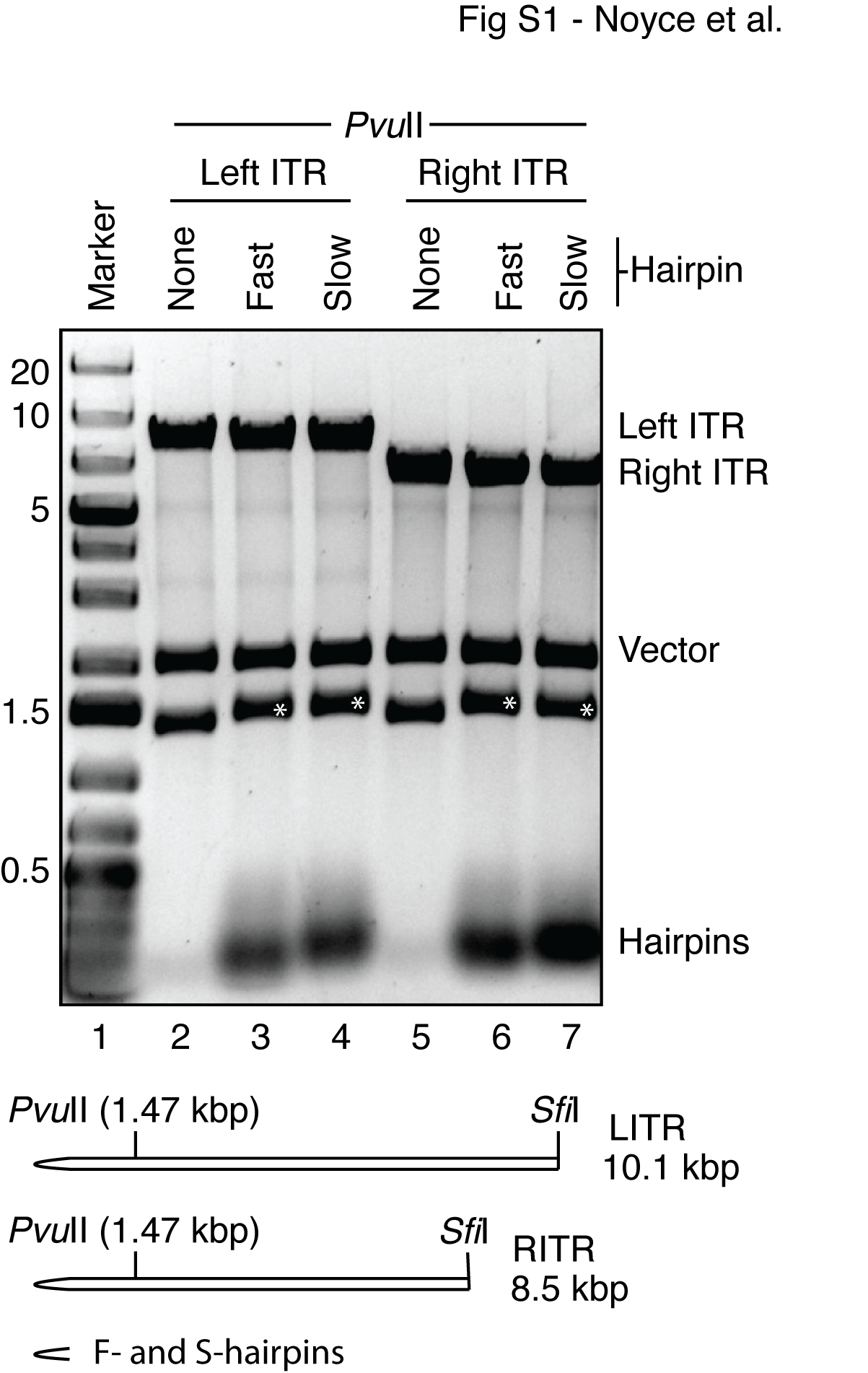

Supplement: S1 Fig — a. Restriction maps showing the arrangement of SfiI and PvuII restriction sites in the left and right ITR clones. b. Gel electrophoretic analysis of ligation products. The cut ITR clones were ligated with or without the fast (F) or slow (S) forms of the VACV hairpin (H.P.) oligonucleotides and then a portion of each sample was digested with PvuII to aid in detecting the small size shift associated with adding hairpin ends. We observed nearly quantitative ligation of the ITR ends to the hairpin oligonucleotides as judged by the bands running at ~1.5 kbp (“*”). (TIF) [file pone.0188453.s002.tif]

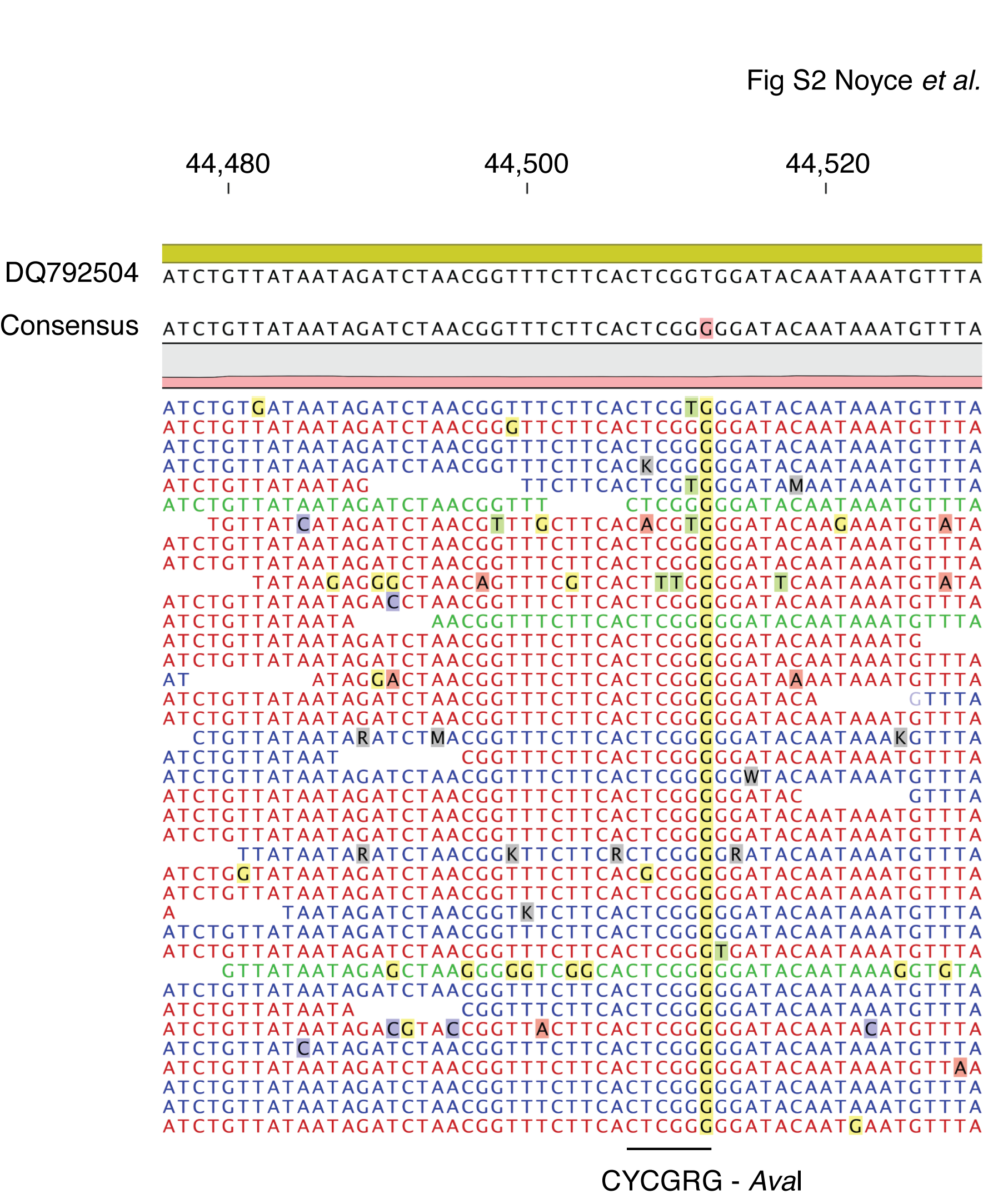

Supplement: S2 Fig — The recovered viruses were sequenced using “Tagmentation” and Illumina technologies and the genomes assembled using CLC genomics software and both “map to reference” and de novo approaches. Shown here are a portion (~1%) of the sequence reads that mapped to the HPXV044 gene (VACV I4L homolog) in DQ792504 (top row). Fragment_2 encoded a silent T-to-G substitution mutation at position 44,512 designed to introduce a novel AvaI site into the reactivated viruses (“Consensus”). Apart from the mutations that were deliberately incorporated into the synthetic sequence, like this T-to-G substitution, no other mutations were detected by genome sequencing across the scHPXV YFP-gpt::095 or scHPXV genomes. (TIF) [file pone.0188453.s003.tif]

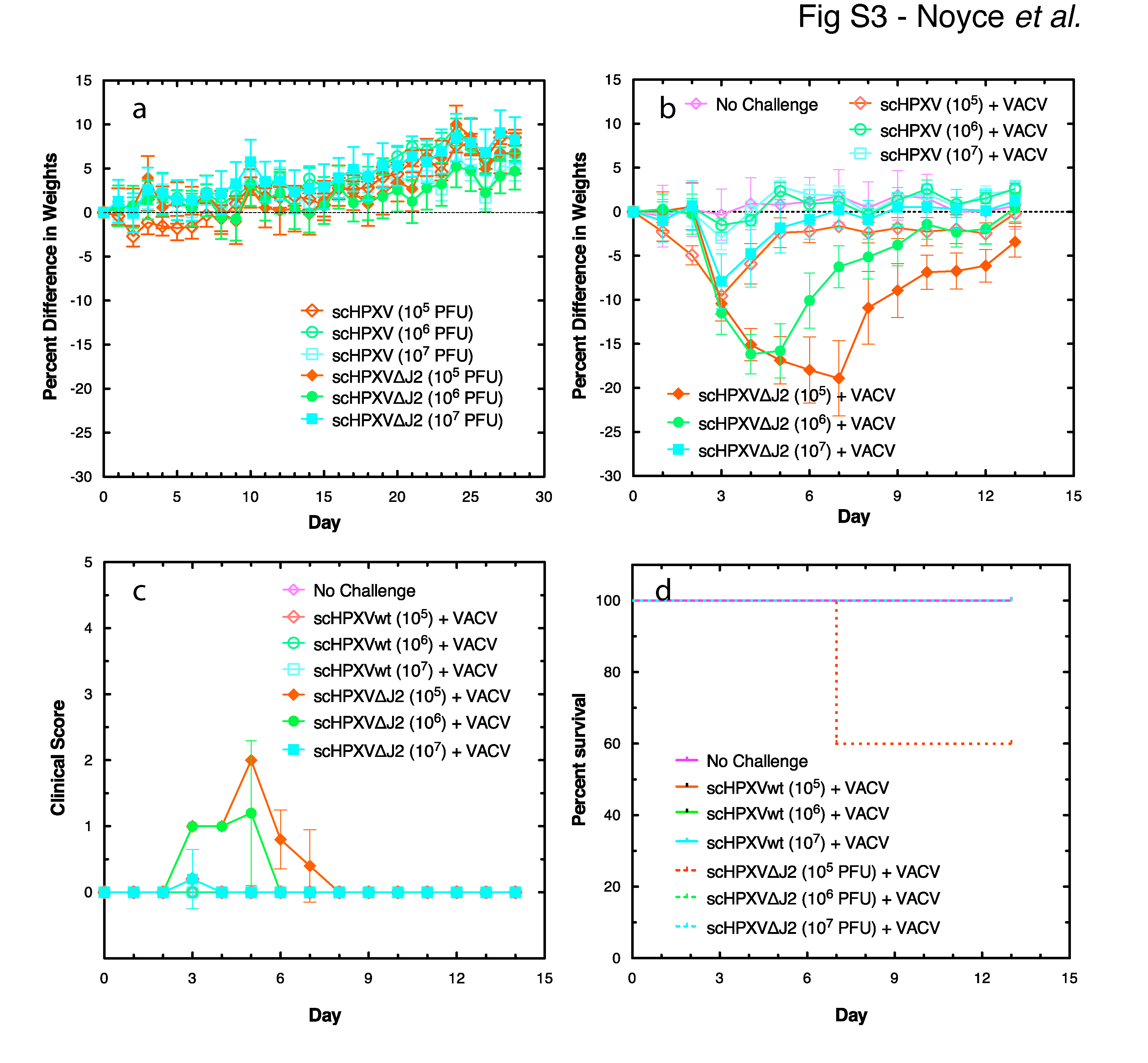

Supplement: S3 Fig — a. Virulence studies. Immune competent BALB/c mice (5 per group) were inoculated intranasally with the indicated viruses. Both scHPXV and scHPXV YFP-gpt::095 (DJ2) were tested, at doses ranging from 105−107 pfu per mouse. No illness was detected in any of the animals infected with HPXV clones. b. VACV challenge studies. Four weeks after exposure to the indicated agents as shown in panel a, the mice were exposed to a lethal dose of VACV strain WR (1×10^6^ pfu) by the same route. (The “no challenge” controls were PBS treated and unchallenged.) The animals were monitored for signs of disease and euthanized if the weight loss exceeded 25% of the initial body weight. The animals previously treated with the scHPXV YFP-gpt::095 strain acquired only limited and dose-dependent protection from VACV strain WR, whereas the scHPXV strain provided good protection at the two highest pre-challenge doses (1×10^6^ and 1×10^7^ pfu). c. Disease course in HPXV-vaccinated mice. The mice were inspected to detect four signs of disease (ruffled fur, hunched posture, difficulty breathing, and reduced mobility) and a clinical score calculated from the sum of the individual scores averaged across all five (or surviving) mice per cohort. Little or no signs of illness were detected in mice first vaccinated with 1×10^6^ or 1×10^7^ pfu scHPXV whereas the scHPXV YFP-gpt::095 provided incomplete protection at the lower pre-challenge doses (1×10^5^ or 1×10^6^ pfu). d. Kaplan-Meyer analysis of survivorship. The lowest dose of scHPXV YFP-gpt::095 (1×10^5^ pfu) provided only partial protection from a VACV challenge. All other interventions protected 100% of the animals. Note that data showing the behavior of the scHPXV strain (presented in Fig 5) are also duplicated here for comparison purposes. (TIF) [file pone.0188453.s004.tif]
