# Supplementary material for: Construction of an infectious horsepox virus vaccine from chemically synthesized DNA fragments
Source: PLoS One. 2018 Jan 19;13(1):e0188453. doi: 10.1371/journal.pone.0188453 (PMC5774680; doi:10.1371/journal.pone.0188453)
Supplement: S1 Table — (DOCX) [file pone.0188453.s005.docx]

**S1 Table. Silent mutations created in scHPXV fragments to remove *Aar*I and *Bsa*I restriction sites from HPXV genome.**

| **HPXV Fragment** | **Restriction endonuclease recognition site removed** | **Nucleotide change in coding strand of HPXV genome** | **HPXV Gene** | **Location of nucleotide change in HPXV genome [DQ792504]** | **Mutation verified by whole genome sequencing** |
| --- | --- | --- | --- | --- | --- |
| Frag_1A | *Bsa*I | A to G | HPXV011a | 11,228 | √ |
| Frag_1B | *Bsa*I | A to G | HPXV025 | 27,845 | √ |
| Frag_2 | *Bsa*I | A to G | HPXV040 | 41,232 | √ |
|  | *Bsa*I | G to A | HPXV059 | 56,775 | √ |
|  | *Bsa*I | G to A | HPXV066 | 67,836 | √ |
| Frag_3 | *Bsa*I | G to A | HPXV083 | 84,361 | √ |
|  | *Aar*I | T to C | HPXV091 | 89,368 | √ |
| Frag_4 | *Bsa*I | T to C | HPXV099 | 96,239 | √ |
|  | *Bsa*I | A to G | HPXV099 | 96,437 | √ |
|  | *Bsa*I | A to G | HPXV110 | 109,492 | √ |
|  | *Bsa*I | A to G | HPXV111 | 110,661 | √ |
|  | *Bsa*I | G to A | HPXV111 | 110,840 | √ |
| Frag_4 Frag_5 | *Bsa*I | C to T | HPXV119 | 120,933 | √ |
| Frag_5 | *Bsa*I | A to G | HPXV123 | 123,035 | √ |
|  | *Bsa*I | T to C | HPXV145 | 144,834 | √ |
| Frag_5 Frag_6 | *Bsa*I | T to C | HPXV146d | 149,727 | √ |
| Frag_6 | *Bsa*I | G to A | HPXV178b | 175,070 | √ |
| Frag_7 | *Bsa*I | G to A | HPXV182 | 180,573 | √ |
|  | *Bsa*I | A to G | HPXV192 | 187,476 | √ |
|  | *Aar*I | G to A | HPXV193 | 188,761 | √ |
|  | *Bsa*I | C to T | HPXV197 | 195,680 | √ |
|  | *Aar*I | T to C | HPXV200 | 199,873 | √ |
