# Supplementary material for: Construction of an infectious horsepox virus vaccine from chemically synthesized DNA fragments
Source: PLoS One. 2018 Jan 19;13(1):e0188453. doi: 10.1371/journal.pone.0188453 (PMC5774680; doi:10.1371/journal.pone.0188453)
Supplement: S2 Table — (DOCX) [file pone.0188453.s006.docx]

**S2 Table. Primers used to amplify regions within VACV and HPXV that flank *Bsa*I restriction endonuclease sites found in VACV (NC_006998).**

| **Synthetic HPXV fragment** | **Primer Name** | **Primer sequence (5’ to 3’)** | **Position of *Bsa*I site in VACV [NC_006998]** | **Position of *Bsa*I site in HPXV [DQ792504]** |
| --- | --- | --- | --- | --- |
| Frag_1A | HPXV 1A - FWD | CTGTATACCCATACTGAATTGATGAAC | 16756 | 27,849 |
|  | HPXV 1A - REV | GAGTTAATATAGACGACTTTACTAAAGTCATG |  |  |
| Frag_1B | HPXV 1B - FWD | GGTTCTTTTTATTCTTTTAAACAGATCAATGG | 23,076 | N/A |
|  | HPXV 1B - REV | TTCTTATTAAGACATTGAGCCCAGC |  |  |
| Frag_2 | HPXV 2A - FWD | AGTCATCAATCATCATTTTTTCACC | 30,073 | 41,225 |
|  | HPXV 2A - REV | ATATAACGGACATTTCACCACC |  |  |
|  | HPXV 2B – FWD | GTAACATATACAACTTTTATTATGGCGTC | 45,485 | 56,778 |
|  | HPXV 2B – REV | CTAATCCACAAAAAATAGAATGTTTAGTTATTTTG |  |  |
|  | HPXV 2C – FWD | AGTGACTGTATCCTCAAACATCC | 56,576 | 67,839 |
|  | HPXV 2C – REV | TTTATAAAGGGTTAACCTTTGTCACATC |  |  |
| Frag_3 | HPXV 3A – FWD | TTGTGTAGCGCTTCTTTTTAGTC | 60,981 | N/A |
|  | HPXV 3A – REV | AAACGGATCCATGGTAGAATATG |  |  |
|  | HPXV 3B – FWD | TATTTGCATCTGCTGATAATCATCC | 84,916 | 84,353 |
|  | HPXV 3B – REV | CGATGGATTCAAATGACTTGTTAATG |  |  |
| Frag_4 | HPXV 4A – FWD | ATGCCTTTACAGTGGATAAAGTTAAAC | 85,101 | 96,243 & 96,428 |
|  | HPXV 4A – REV | CTGGATCCTTAGAGTCTGGAAG |  |  |
|  | HPXV 4B – FWD | CGGAAAATGAAAAGGTACTAGATACG | 98,134 | 109,485 |
|  | HPXV 4B – REV | TGAATAGCCGTTAAATAATCTATTTCGTC |  |  |
|  | HPXV 4C – FWD | TATGGATACATTGATAGCTATGAAACG | 99,302 & 99,481 | 110,653 & 110,832 |
|  | HPXV 4C – REV | AATACATCTGTTAAAATTGTTTGACCCG |  |  |
| Frag_5 | HPXV 5A – FWD | CATTTTATTTCTAGACGTTGCCAG | 111,686 | 123,037 |
|  | HPXV 5A – REV | CGATATGAAACTTCAGGCGG |  |  |
|  | HPXV 5B – FWD | ACAAAACGATTTAATTACAGAGTTTTCAG | 122,484 | N/A |
|  | HPXV 5B – REV | GTCCGGTATGAGACGACAG |  |  |
|  | HPXV 5C – FWD | TTAGGGATCACATGAATGAAATTCG | 133,505 | 144,838 |
|  | HPXV 5C – REV | TATGGAAGTTCCGTTTCATCCG |  |  |
|  | HPXV 5D – FWD | GACTTGATAATCATATATTAAACACATTGGATC | 138,306 | 149,718 |
|  | HPXV 5D – REV | AGATCTCCAGATTTCATAATATGATCAC |  |  |
| Frag_6 | HPXV 6A – FWD | ATGATACGTACAATGATAATGATACAGTAC | 163,521 | 175,062 |
|  | HPXV 6A – REV | TGATTTTTGCAATTGTCAGTTAACACAAG |  |  |
| Frag_7 | HPXV 7A – FWD | TACTGTACCCACTATGAATAACGC | 169,035 | 180,578 |
|  | HPXV 7A – REV | GATATCAACATCCACTGAAGAAGAC |  |  |
|  | HPXV 7B – FWD | ATCTTACCATGTCCTCAAATAAATACG | 175,849 | 187,467 |
|  | HPXV 7B – REV | ATAGCTCTAGGTATAGTCTGCAAG |  |  |
|  | HPXV 7C – FWD | GCGAACTCCATTACACAAATATTTG | 181,952 | 195,683 |
|  | HPXV 7D - REV | GATGTTTCTAAATATAGGTTCCGTAAGC |  |  |
