# Supplementary material for: Construction of an infectious horsepox virus vaccine from chemically synthesized DNA fragments
Source: PLoS One. 2018 Jan 19;13(1):e0188453. doi: 10.1371/journal.pone.0188453 (PMC5774680; doi:10.1371/journal.pone.0188453)
Supplement: S3 Table — (DOCX) [file pone.0188453.s007.docx]

| **S3 Table. Read statistics of F- and S- hairpins (HP) in reactivated scHPXV clones.** | | | | |  |  |  |  |
| --- | --- | --- | --- | --- | --- | --- | --- | --- |
| **Sample** | **Total Reads** | **Map to scHPXV YFP-gpt::095 (no hairpin)** | | **Map to F hairpin only** | | **Map to S hairpin only** | | **Ratio of mapped F to S reads** |
|  |  | **Mapped Reads** | **Average**  **Coverage** | **Mapped Reads** | **Average Coverage** | **Mapped**  **Reads** | **Average Coverage** |  |
| **scHPXV YFP-gpt::095 (1-1) - R1**^a^ | 3405278 | 3306669 | 3149 | 384 | 345 | 375 | 355 | 1.02 |
| **scHPXV YFP-gpt::095 (2-1) R1** | 4015666 | 3922329 | 3463 | 392 | 343 | 375 | 359 | 1.05 |
| **scHPXV YFP-gpt::095 (3-1) - R1** | 2137262 | 2078853 | 1991 | 106 | 90 | 101 | 94 | 1.05 |
| **scHPXV YFP-gpt::095 (1-1) - R2** | 5093520 | 5012818 | 5370 | 117 | 101 | 115 | 102 | 1.02 |
| **scHPXV YFP-gpt::095 (3-1) - R2** | 3908448 | 3835013 | 4357 | 132 | 110 | 126 | 119 | 1.05 |
| **scHPXV (1-1)** | 3756246 | 3594301 | 4062 | 161 | 150 | 166 | 148 | 0.97 |
| **scHPXV (2-1)** | 4790068 | 3396971 | 3576 | 151 | 127 | 144 | 138 | 1.05 |
| **scHPXV (3-1)** | 4737982 | 4468229 | 4929 | 228 | 200 | 216 | 212 | 1.06 |

^a^R1 and R2 refer to independent HPXV reactivation reactions.
